# A Novel Intelligent Rebound Hammer System Based on Internet of Things

**DOI:** 10.3390/mi14010148

**Published:** 2023-01-06

**Authors:** Zongqiang Pang, Qing Wang, Yong Wang, Zhiyin Gong

**Affiliations:** College of Automation & College of Artificial Intelligence, Nanjing University of Posts and Telecommunications, Nanjing 210003, China

**Keywords:** rebound hammer, internet of things, laser ranging, phonetic transcription, cloud platform

## Abstract

In order to improve the test efficiency of concrete strength and ensure measured data reliability, we present a novel intelligent rebound hammer system which is based on the Internet of Things (IoT) and speech recognition technology. The system uses a STM32F103C8T6 microcontroller as the Main Control Unit (MCU), and one BC26 module as the communication unit, combined with a LD3320 voice recognition module and TOF050H laser ranging sensor to achieve the function of phonetic transcription and laser ranging. Without the need for traditional multi-person collaboration and burdensome data transfer, the system can collect the data of rebound value and location information and send them to the remote cloud information management system automatically in real time. The test results show that the system has high measuring accuracy, good data transmission stability and convenient operation, which could provide guidance for other types of non-destructive testing equipment designs.

## 1. Introduction

The strength of concrete is related to the safety of the main structure of a building; it is the most important part of quality control of a cast-in-place concrete structure. However, during the actual construction process, construction safety accidents often occur due to misoperation by front-line staff or inadequate supervision from managers, both of which have caused huge economic losses. The testing methods of concrete strength can be divided into destructive and nondestructive testing. The destructive testing method, which involves drilling a cylindrical sample from the main structure and testing it inside the laboratory, has higher measurement accuracy but comes with high costs and is time-consuming. Most importantly, the method may cause irreparable damage to the concrete structure, which makes it unusable for some important concrete structures or other important projects with special requirements.

The nondestructive testing method does not impair the building structure and allows for re-testing at the same location to compare the changes in concrete quality. As the concrete is susceptible to a variety of environmental degrading factors which tend to limit its service life, concrete strength should be tested in situ periodically for quality assurance and the evaluation of existing conditions [1]. Among the many nondestructive testing methods, such as ultrasonic pulse velocity [2], ray absorption and scattering, the rebound hammer and the ultrasonic-rebound combined method, the rebound hammer is deemed as the most popular method for concrete quality testing, which is popular for its simple structure, ease of use and low cost [3,4]. The basic principle of the rebound hammer is to use the spring-driven heavy hammer to make the heavy hammer hit the impact bar that is in vertical contact with the concrete surface with constant kinetic energy in order that the local concrete deforms and absorbs part of the energy. The other part of the energy will be converted into the rebound kinetic energy of the heavy hammer. When all the rebound kinetic energy is converted into potential energy, the heavy hammer will rebound to a maximum distance. The rebound number is the ratio of the maximum springback distance to the initial spring length. According to the JGJ/T 23-2011, the rebound hammer uses the rebound number which is related to the surface hardness of the concrete to evaluate the strength of the concrete [5].

Most early rebound hammers involve contact measuring and require manual reading and recording of its rebound value. Due to the friction of the pointer during application, some metal powder may attach to the resistors, which will affect the sensitivity of the sensor, so this type of rebound hammer has lower efficiency and a short lifespan [6]. In order to settle the friction problem, Shejiao’s team developed one intelligent rebound hammer system which uses linear CCD imaging as the non-contact rangefinder sensor to measure the displacement of the hammer; the MCU is used for circuit driving and image processing back in the office after the test and transfers all the measured data to the computer through a USB port [7]. In 2007, they developed another intelligent rebound hammer system which was based on PSD and Zigbee technology and which achieved non-contact range-finding and wireless data transmission [8]. However, for the location information of the test area, it still requires another surveyor to record synchronously. Moreover, limited by weak diffraction, weak signal penetration and the requirement of an ad hoc network for Zigbee technology, it is not convenient for use in actual construction sites [9].

In this paper, we present one novel intelligent rebound system which is based on IoT and speech recognition technology [10]. Using laser ranging sensor to measure the hammer displacement and a voice recognition module to record the location information under testing, the MCU collects the data and sends them to the remote cloud information management system by the IoT module in real time [11]. There is no need for traditional multi-person collaboration and burdensome data transfer. Most importantly, it avoids the risks of artificial modification and entry error of the test date, which seriously affect the quality and safety of the constructions.

## 2. System Design

**Figure 1:**
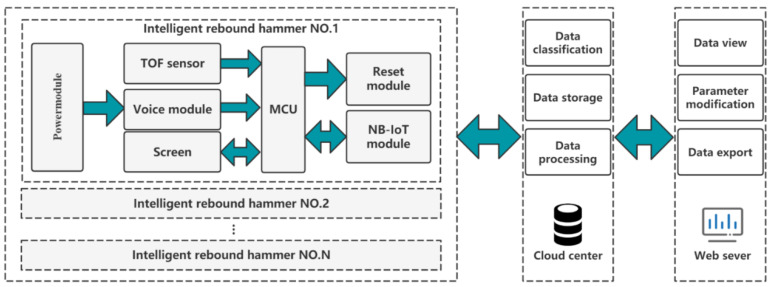
The schematic diagram of system structure.

The intelligent rebound system should have the functions of hammer displacement measuring, phonetic transcription, real-time data remote transmission, data storage and analysis, etc [12]. Among those elements, the phonetic transcription unit is required to complete the operator’s voice recognition and transcription of location information, such as “101 East Wall Area 1” and “203 North Wall Area 4”. The real-time data remote transmission unit is responsible for sending the hammer rebound value and phonetic transcription data to the cloud server in real time through the IoT module. The cloud information management unit is responsible for storing the data information transmitted by IoT and responding to the web request to render the data to the front-end page [13]. The schematic diagram of the system structure is shown in Figure 1.

### 2.1. Circuit Design

With consideration for the number of communication interfaces, the size of the hardware circuit board and the expansion space for subsequent system upgrading [14], the STM32F103C8T6 microcontroller is selected as the system MCU, which has up to 37 digital input/output ports, two 12-bit synchronized ADCs, two IICs, three UARTs, two SPIs, one CAN, three 16-bit timers, and 64 KB of program memory. The whole system is powered by a 5 V rechargeable lithium-ion battery pack with a capacity of 8400 mAh, which can work stably for longer than 8 h without interruption.

For non-contact range-finding, we chose a TOF050H module as the ranging sensor to measure the hammer rebound value, which works under 3.3 V and supports serial-mode, modbus-mode and IIC-mode data transfer [15]. In view of the range of rebound value [16], the high precision ranging mode is selected, the module range is 200 mm, accuracy is ±5%, resolution is 1 mm, and the ranging period is set to 200 ms.

For phonetic transcription, we chose a widely used LD3320 module to recognize and transcript the voice information of different locations, which has high-precision A/D and D/A interfaces and can achieve phonetic transcription without additional external auxiliary Flash and RAM [17]. During voice signal processing, the environment noise will firstly be removed from the recognized voice signal. The SPI clock, chip select and input and output pins are connected to the external MCU by SPI protocol to achieve serial interface communication. In view of the limited number of offline entries, the method of word combination is adopted to expand the recognition capability of LD3320, which uses the external MCU to sort and integrate recognized entries according to the time series.

**Figure 2:**
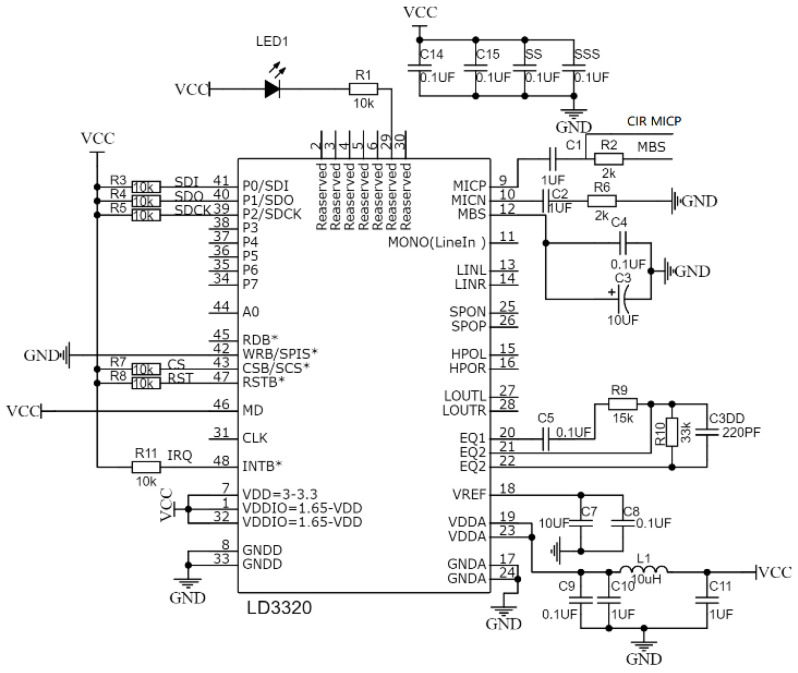
The schematic diagram of phonetic transcription unit.

In order to improve the recognition rate, an RC circuit is added to the microphone bias pin (MBS) to ensure a floating voltage is applied on the microphone. The schematic diagram of phonetic transcription unit is shown in in Figure 2.

For data remote transmission, we chose a Quectel BC26 communication module to send the hammer rebound value and phonetic transcription data to the cloud server in real time, which has low power consumption and high performance [18]. The BC26 module communicates with MCU through a UART serial port and builds a connection with the cloud server by TCP protocol [19].

**Figure 3:**
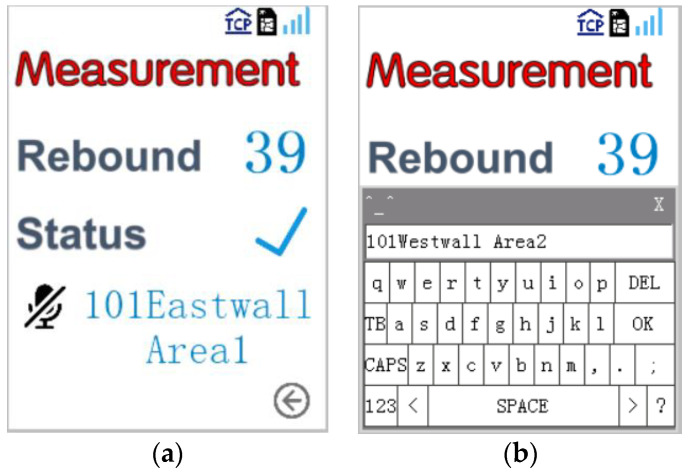
(**a**) The user interface screen of intelligent rebound hammer. (**b**) The screen of user interface during modification of the recognized speech text.

In order to check the phonetic transcription results and the working status of the intelligent rebound hammer in real time, a 2.4-inch touchscreen display was selected as the user interface (TJC3224T124_011R, Taojingchi, Shenzhen, China); it works under 5 V and communicates with MCU through a serial port. The measured rebound value, recognized location information, communication signal strength and sending status are displayed on the display screen. If any errors in phonetic transcription are found, the operator can modify the speech text by the screen keyboard and send the revised text to the external MCU through serial port interrupt, as shown in Figure 3.

### 2.2. Structure Design

**Figure 4:**
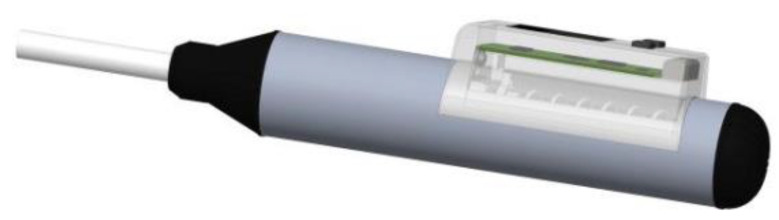
The schematic view of intelligent rebound hammer.

In view of the ranging characteristics of the TOF050H module, the white reflector board is fixed on the slider, which can slide along the sliding rail. The nominal kinetic energy of the hammer is 2.207 J, and the measuring strength range of 10~60 MPa is related to the rebound displacement range of 20~60 mm. In view of the location and field angle of the ranging sensor, the width of the reflector board is set to 17 mm, which ensures the ranging sensor can cover the full field. The touchscreen is fixed on the top of the circuit box, and a 5.0 V rechargeable polymer lithium battery, which provides a power source for the rebound hammer, is fixed inside the circuit box. The schematic view of the intelligent rebound hammer is shown in Figure 4.

### 2.3. MCU Program Design

In order to distinguish the rebound value and phonetic transcription data from different intelligent rebound hammers, an 8-bit array is set to store the rebound value in which the front two bits are the data category, the middle four bits are the equipment number, and the last two bits are the rebound value. For example, if rebound hammer #1 is used to measure the concrete strength and the rebound value is 40 mm, the uploaded array will be “00000140”. Furthermore, another 12-bit array is set to store the phonetic transcription data in which the front two bits are the data category, the middle four bits are the equipment number, and the last six bits are the phonetic transcription. 

**Figure 5:**
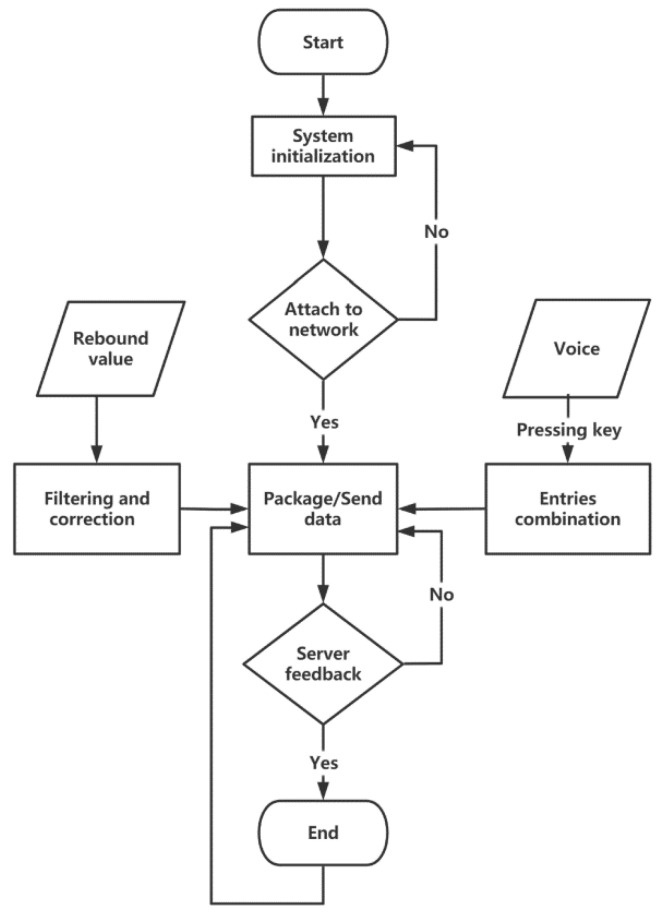
The program flow chart of the MCU.

When the system is turned on, the system starts initializing, the BC26 module connects to the network. After network attaching, when the BC26 module closes the previous TCP connection and creates a new TCP connection, the system enters the main loop, which includes phonetic transcription, range-finding and data remote transmission. If the voice recognition button is held down, the system enters the process of voice recognition until the release of the button. The MCU sorts and integrates all recognized entries according to the time series, and the final result of phonetic transcription is sent to the cloud server as a 12-bit array. The MCU sends the rebound value and phonetic transcription to the server by calling the AT instruction and checks the returned value to judge the sending status. The program flow chart is shown in Figure 5.

### 2.4. Web Program Design

After establishing the connection with the server, the rebound hammer sends the collected data to a specified port. The server listens to the port via Python scripts, then analyzes, classifies and stores the received data into the Mysql database. The received 12-bit array will be translated into a text of location information according to the hash table, whereas the received 8-bit array of rebound values will be bound to the location according to the category and equipment number. 

**Figure 6:**
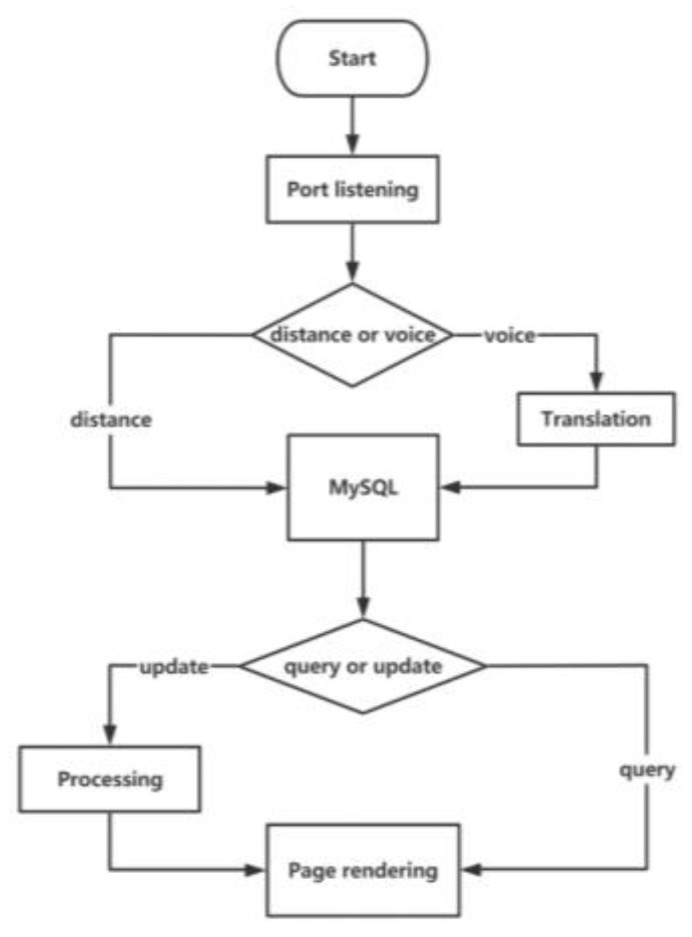
The workflow chart of the cloud information management platform.

The front end of the cloud information management platform is developed by Vue.js, and the back end is developed by Python language. In view of the development cycle and extensibility, the web server is built with a Flask framework and interacts with the database via a PyMySQL module [20]. If the web server receives any requests from the front-end interface, it will render the response results to the browser. The workflow chart is shown in Figure 6.

**Figure 7:**
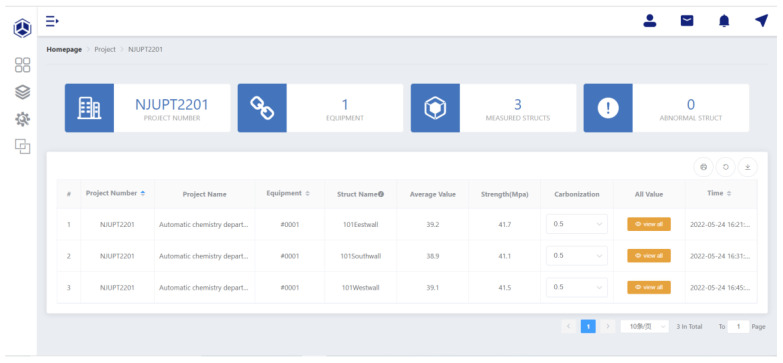
The webpage of the cloud information management platform.

As shown in Figure 7, on the webpage of the cloud information management platform, the manager can check the received test data, modify related parameters and export them to a text or excel file. After the parameter modification, such as the modification of impact angles, pouring surface and carbonization depth, the back end of the platform will modify the rebound value according to the technical regulations and convert the rebound value to the strength of the concrete automatically according to “Conversion Table of Compressive Strength of Concrete for Test Area”. 

## 3. Experimental Results

**Figure 8:**
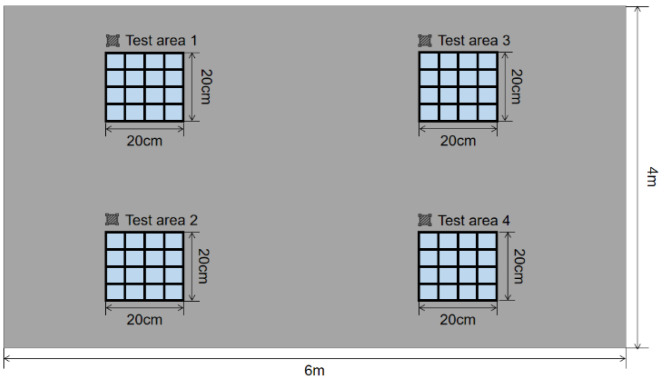
The schematic view of the measuring points for four test areas.

In order to check the performance of our home-made intelligent rebound hammer system, we chose one state grid substation construction in Nanjing to test the concrete strength. As shown in Figure 8, we chose four test areas on one load-bearing wall arbitrarily and divided them into 4 × 4 small grids, respectively. According to the JGJ/T 23-2011, before the test, the calibrated steel anvil of 60 ± 2 Rockwell hardness was used to calibrate the two rebound hammers, and the calibration results were within 80 ± 2. With the home-made intelligent rebound hammer, we tested the concrete strength for each small grid. The platform receives 16 rebound values for each test area, deletes 3 maxima and 3 minima, and finally calculates an average rebound value for each test area. In order to compare the performance, we chose another four close test areas in the same load-bearing wall and one commercial digital rebound hammer (YD225P, Daolong Technology Co., Ltd., Shenzhen, China). The test value of YD225P is deemed as the standard value, and the impact angle is set to 0. The test results of four test areas are shown in Table 1.

From Table 1, the relative error between two rebound hammer test results for four test areas is 0%, 0.70%, 0%, and 1.50%, respectively, which proves the good performance of our home-made intelligent rebound hammer system. After hundreds of tests and comparative analyses on different concrete structures in several engineering sites, we have confirmed the working performance and stability of our intelligent rebound hammer system. Most importantly, it can send the data of rebound value and location information to a remote cloud information management system without traditional multi-person collaboration, which avoids the risks of artificial modification or data transfer errors.

## 4. Conclusions

We have presented one novel intelligent rebound hammer based on IoT and speech recognition technology, which use a TOF050H laser ranging sensor to measure the hammer displacement and a LD3320 voice recognition module to record the location information. All the test data will be sent to a remote cloud information management system by the IoT module in real time, which can solve the problems of multi-person collaboration and burdensome data transfer and consequently avoid the risks of artificial modification and data transfer errors.

## Figures and Tables

**Table 1:** Test Results of Four Test Areas.

Table 225.	YD225C					Home-Made					Relative Error
	Test Value				Mean Value	Test Value				Mean Value	
1	40	39	38	25	38.7	39	39	38	36	38.7	0%
	38	23	53	41		38	39	39	39		
	38	39	53	34		39	39	39	40		
	39	39	39	38		37	37	38	39		
2	24	39	39	40	39.4	41	39	39	40	39.7	0.70%
	40	40	40	39		40	39	24	40		
	39	39	42	38		40	39	54	54		
	39	40	40	39		40	26	42	35		
3	39	39	38	37	37.7	37	25	40	38	37.7	0%
	36	35	37	36		37	36	29	38		
	38	37	41	39		37	39	35	40		
	40	38	23	38		37	40	38	41		
4	27	38	26	39	39.1	40	38	39	39	38.5	1.50%
	26	39	39	40		38	38	38	39		
	41	39	38	40		37	40	33	37		
	44	46	42	38		39	39	39	38		

## Data Availability

The authors are unable or have chosen not to specify which data have been used.
